# Cell Kinetics of Hepatic Metastases as a Prognostic Marker in Patients With Advanced Colorectal Carcinoma

**DOI:** 10.1155/1990/12854

**Published:** 1990

**Authors:** Rosella Silvestrini, Aurora Costa, Leandro Gennari, Roberto Doci, Emilio Bombardieri, Luigia Bombelli

**Affiliations:** 1 Surgical Oncology A lstituto Nazionale per lo Studio e la Cura dei Tumori Via Venezian Milan 1-20133 Italy; 2 Nuclear Medicine Service lstituto Nazionale per lo Studio e la Cura dei Tumori Via Venezian Milan 1-20133 Italy; 3 Istituto Nazionale Tumori Via Venezian 1 Milan 20133 Italy

## Abstract

Cell kinetics was determined, as ^3^H-thymidine labeling index (LI), in hepatic lesions from 36 patients with primary colorectal carcinoma; LI values ranged from 0.9% to 23.5% and were normally distributed. Cell kinetics was not related to sex or age of the patient, or to liver function. For clinical studies the median LI value of 10% was used to separate slowly and rapidly proliferating lesions. Univariate analysis showed that patients radically resected and with a low LI tumor have a longer disease-free interval and a better probability of 12-month survival than those non-radically resected and with a high LI tumor. When treatment and cell kinetics were taken into consideration, the probability of 12-month survival was 100% for patients with slowly proliferating and radically resected hepatic metastases. Patients with rapidly proliferating tumors, regardless of type of treatment, had the worst prognosis.


*HPB Surgery* 1990, Vol. 2 pp. 135–144
Reprints available directly from the publisher
Photocopying permitted by license only

© 1990 Harwood Academic Publishers GmbH
Printed in the United Kingdom

### CELL KINETICS OF HEPATIC METASTASES AS A PROGNOSTIC MARKER IN PATIENTS WITH ADVANCED COLORECTAL CARCINOMA

ROSELLA SILVESTRINI*, AURORA COSTA, LEANDRO GENNARI,* ROBERTO DOCI*, EMILIO BOMBARDIERI, and LUIGIA BOMBELLI*

**Experimental Oncology C, *Surgical Oncology A and Nuclear Medicine Service. Istituto Nazionale per lo Studio e la Cura dei Tumori Via Venezian, 1 – 20133 Milan, Italy*

*(Received 21 March 1989)*

Cell kinetics was determined, as $^{3}H$-thymidine labeling index (LI), in hepatic lesions from 36 patients with primary colorectal carcinoma; LI values ranged from 0.9% to 23.5% and were normally distributed. Cell kinetics was not related to sex or age of the patient, or to liver function. For clinical studies the median LI value of 10% was used to separate slowly and rapidly proliferating lesions. Univariate analysis showed that patients radically resected and with a low LI tumor have a longer disease-free interval and a better probability of 12-month survival than those non-radically resected and with a high LI tumor. When treatment and cell kinetics were taken into consideration, the probability of 12-month survival was 100% for patients with slowly proliferating and radically resected hepatic metastases. Patients with rapidly proliferating tumors, regardless of type of treatment, had the worst prognosis.

KEY WORDS: Cell kinetics, hepatic metastases, colorectal cancer, prognosis

### INTRODUCTION

Hepatic metastases in patients with colorectal carcinoma always represent a poor prognostic factor for patient survival. However, the clinical course varies from a few months to some years.

Various classifications have been proposed[1–8], and biologic markers and pathologic features indicative of risk have been analyzed in multivariate analyses and ranked according to their relative ability to predict survival[9,10,11].

Cell kinetics, a variable of prognostic relevance already proved in some human tumor types, has been determined in primary colorectal tumors[12–18], but it has never been investigated in hepatic metastases. With the present study, we proposed to define the potential proliferative activity of hepatic metastases from colorectal carcinoma, in the different clinico-pathologic situations, and to investigate its prognostic relevance and its relative importance compared with conventional factors in predicting clinical outcome.

*Address reprint requests to: Rosella Silvestrini, Ph.D., – Istituto Nazionale Tumori, Via Venezian 1, 20133 Milan, Italy.

### PATIENTS AND METHODS

From November 1984 to September 1986, 36 patients with hepatic metastases from colorectal adenocarcinoma underwent laparotomy to perform liver resection or positioning of an intraarterial catheter for intrahepatic chemotherapy at the Istituto Nazionale Tumori of Milan. There were 15 females and 21 males, whose ages ranged, at the time of liver resection, from 28 to 70 years (mean, 53 years). In 17 patients the primary was in the colon, in 12 patients in the sigmoid, and in 7 patients in the rectum. Fifteen were synchronous lesions and 21 were metachronous to resection of the primary colorectal tumor.

All patients were evaluated by echotomography, computerized axial tomography (CT), and angiography of the arterial and venous systems of the liver. The patients were staged according to the classification proposed by Gennari *et al.*[1]: the extent of liver replacement was defined as $H_1$, involvement less than 25%; $H_2$, from 25% to 50%; and $H_3$, more than 50%. Biochemical determinations of alkaline phosphatase (AP) and carcinoembryonic antigen (CEA) serum levels were performed at the time of diagnosis.

Twelve patients underwent radical resection of hepatic metastases. In 24 the bilateral extent of metastatic disease in the liver or the presence of extrahepatic intraabdominal growth did not allow surgical treatment. Three of them had debulking surgery plus intraperitoneal administration of 5- fluorouracil. In 11 an intraarterial infusion device was implanted to allow continuous infusion chemotherapy with floxuridine. In the others systemic chemotherapy with 5–fluorouracil was given.

Follow-up studies included CEA assay, liver function tests, echotomography and/or CT every 2–3 months. In the absence of suspected local progression of the disease, a chest roentgenogram was performed at 4–6 month intervals.

Cell kinetics was determined on fresh tumor material immediately after surgery. Tumor tissue, after removal of necrotic areas, was minced into fragments of a few millimeters: 5–10 fragments were incubated in McCoy's 5A medium modified with 20% fetal calf serum, antiobiotics (100 IU of penicillin and 100g of streptomycin/ml) and 6 mCi/ml of $^3$H- thymidine (s.a., 5 Ci/mmol) for 1 h in a Dubnoff shaker bath at 37°C. After incubation, the samples were fixed in Bouin's solution, dehydrated in alcohol and embedded in paraffin. Autoradiography was performed on histologic sections according to the stripping film (Kodak AR10, London, U.K.) technique. After an exposure time of 10 days at 4°C, autoradiograms were developed in Kodak D19b for 5 min at 18°C and fixed in Kodak F5. The samples were stained with hematoxylin and eosin at 4°C[19]. Proliferative activity was determined by scoring a total of 3,000 to 10,000 tumor cells for each tumor and was expressed as $^3$H–thymidine labeling index (LI). The median LI value of 10%, defined for the overall series, was used as the cutoff point to define slowly and rapidly proliferating tumors.

Nonparametric statistics, Wilcoxon and Kruskall-Wallis tests were used to compare the cell kinetic pattern of different subtypes. All hypothesis tests were two sided. Freedom from progression (FFP) was considered as the interval between the start of treatment and the first documented evidence of treatment failure. The actuarial life table method was used to summarize FFP and overall survial distributions, and the statistical significance of differences observed was assessed by the logrank test[20]. The median time of observation for the overall series of patients was 10 months. The percentage of progression-free or live patients was reported for one point in time (12 months) as derived from life-table plots.

### RESULTS

LI values, recorded on the overall series, showed a normal distribution with an interpatient variability from 0.9% to 23.5% and a median LI value of 10% (Figure 1). Cell kinetics was not related to sex or patient's age, and similar spectra of LI values were observed irrespectively of the site of the primary tumor as well as for synchronous and metachronous lesions (Table 1). Median LI values were similar in patients with surgically resectable or non-resectable metastases.

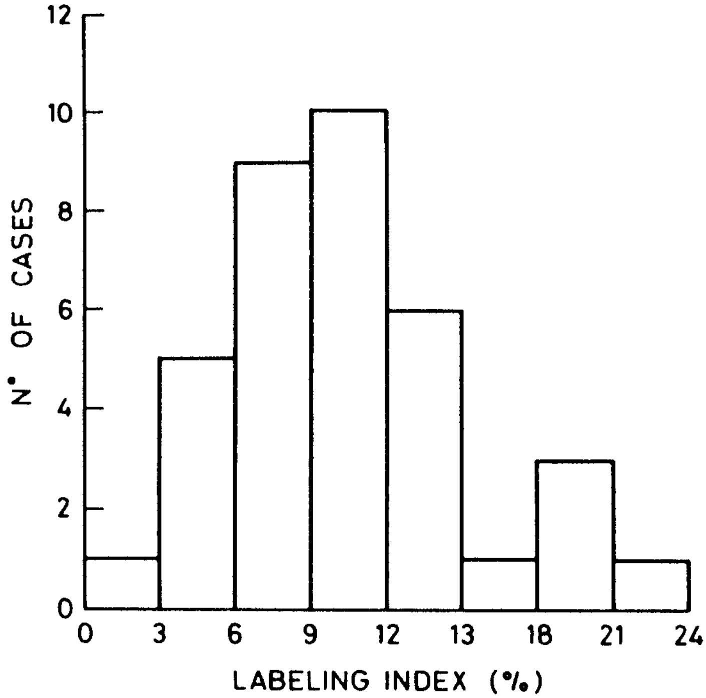

**Figure 1** Frequency distribution of labeling index values of hepatic metastases from colorectal adenocarcinoma.

Cell kinetics of hepatic lesions were not related to CEA levels determined before hepatic resection (data not presented). Similarly, broad LI ranges and no significantly different median values were found for patients with pretreatment low or high alkaline phosphatase levels by using as a cutoff value 279 IU, according to Szasz test (9.3% vs 11.6%) (Figure 2). Conversely, an overall trend of a direct relation, although not statistically significant, was observed between LI values and degree of liver involvement, this finding was limited to synchronous metastases (Table 2).

Various biologic, pathologic and treatment variables have been individually analyzed as predictors of FFP and survival at 12 months for the 35 patients with available follow-up information (Table 3). The median LI of hepatic lesions for this series of patient was 10%, and it included 16 patients with slowly proliferating and 19 patients with rapidly proliferating metastases. The probabilities of FFP and survival for the overall series were 28 and 56%, respectively, in agreement with the data reported from clinical studies on large series of patients. Univariate analysis showed that outcome of surgery, cell kinetics and time of diagnosis of metastases were significant indicators of FFP, with the highest relevance for radical surgery. Radical resection still represented an important discriminant of survival, even though cell kinetics appeared somewhat superior in predicting long-term clinical outcome, whereas time of diagnosis of metastases lost its relevance as an indicator of survival.

**Table 1** Cell kinetics of hepatic metastases in relation to characteristics of the patients, primary tumor and metastases

| | *No. of cases* | *Labeling index (%)* | |
|---|---|---|---|
| | | *Median* | *Range* |
| Sex | | | |
| Male | 21 | 9.5 | 3.7–18.3 |
| Female | 15 | 11.2 | 0.9–23.5 |
| Age (yrs) | | | |
| <50 | 13 | 8.5 | 3.4–23.5 |
| >50 | 23 | 10.0 | 0.9–20.0 |
| Primary Lesion | | | |
| Colon | 17 | 10.4 | 0.9–16.6 |
| Sigmoid-colon | 12 | 9.6 | 3.7–23.5 |
| Recrum | 7 | 10.9 | 3.4–18.3 |
| Metastases | | | |
| Synchronous | 15 | 10.4 | 3.7–23.5 |
| Metachronous | 21 | 9.8 | 0.9–13.8 |

**Table 2** Cell kinetics of hepatic metastases in relation to proposed clinical staging

| | *No. of cases* | *Median labeling index (%)* | | |
|---|---|---|---|---|
| | | *Overall* | *Synchronous metastases* | *Metachronous metasteses* |
| $H_1$ | 16 | 9.4 | 9.4 | 8.6 |
| $H_2$ | 12 | 10.4 | 10.4 | 9.3 |
| $H_3$ | 8 | 14.1 | 17.1 | 10.8 |

The relative prognostic relevance of treatment and cell kinetics, as the two most important prognostic factors for this series of patients, was analyzed. Bivariate analysis showed that cell kinetics was an additional significant discriminant of survival within a group of patients with resectable metastases (Figure 3). In fact, all patients with low LI hepatic lesions were alive at 12 months. Conversely, only about one-third of patients with high LI metastases have a probability to survive at that time. Median time to progression (TTP) was not reached in the former subgroup, and it was 6 months in the

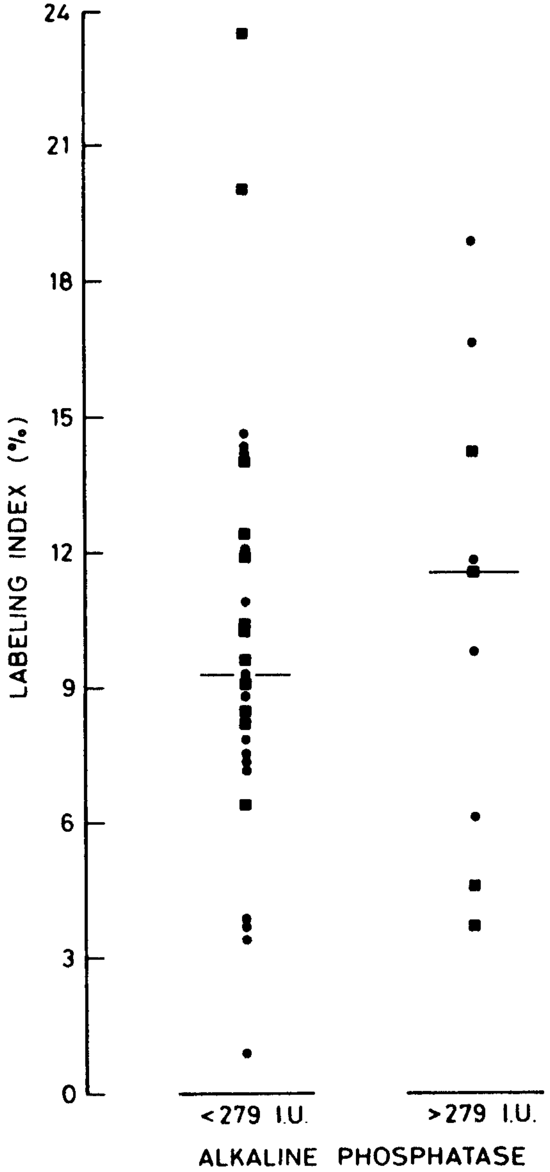

**Figure 2** Scattergrams of labeling index values in relation to levels of alkaline phosphatas ( ■ synchronous lesions; ● metachronous lesions).

latter. In patients with unresectable metastases, median TTP and survival rate at 12 months for patients with low LI lesions were double those observed for patients with high LI lesions. The difference was not statistically different (Figure 4).

The analysis of FFP and survival rates within homogeneous cell kinetic subgroups failed to demonstrate significant differences between patients with resectable and those with unresectable metastases except for a trend in survival of patients with low LI hepatic metastases (Table 4).

**Table 3** Clinical outcome as a function of clinical, pathologic or biologic variables

| | *Probability (%) at 12 mo* | | | |
|---|---|---|---|---|
| | *FFP** | *p* | *Survival* | *p* |
| Treatment | | | | |
| Radical surgery | 56 | <0.01 | 78 | 0.05 |
| Nonradical surgery | 7 | | 43 | |
| Cell kinetics | | | | |
| Low LI | 34 | 0.04 | 79 | <0.01 |
| High LI | 17 | | 22 | |
| Metastases | | | | |
| Metachronous | 37 | 0.02 | 72 | ns |
| Synchronous | 7 | | 36 | |

*FFP: Freedom from Progression

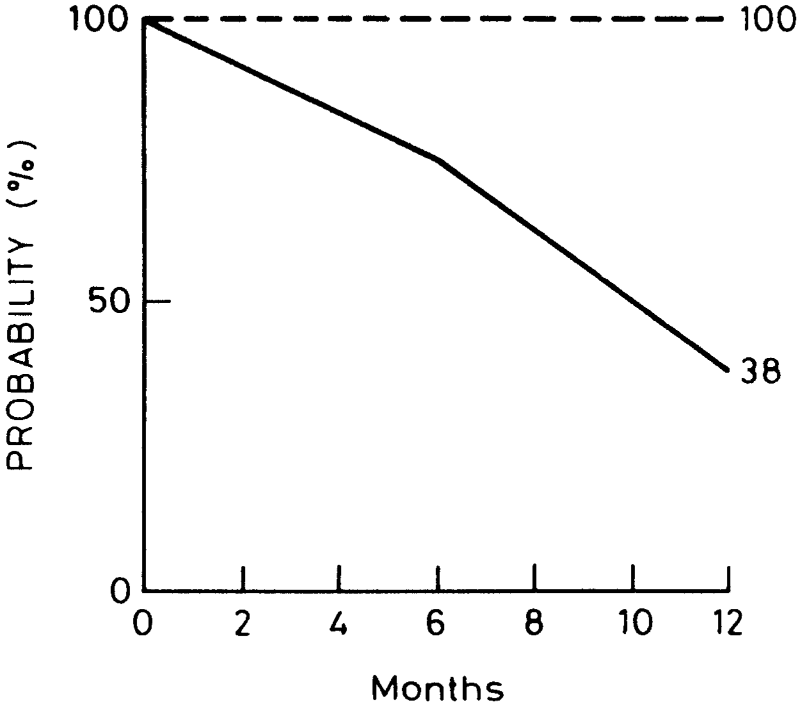

**Figure 3** Actuarial survival of patients with hepatic metastases radically resected. Broken line, low labeling index (≤ 10%); solid line, high labeling index(> 10%). The difference between the two curves was statistically significant (p = 0.03).

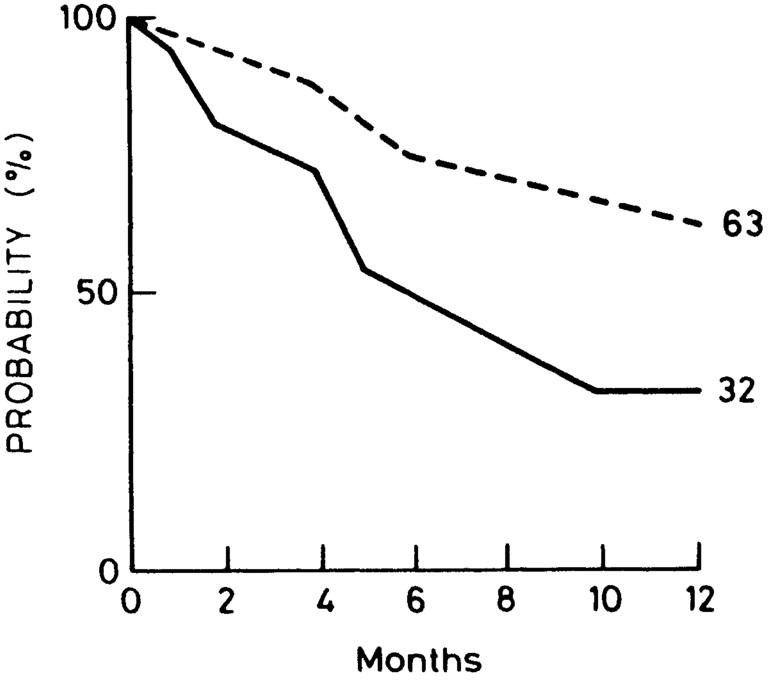

**Figure 4** Actuarial survival of patients whose hepatic metastases were non-radicallyresected. Broken line, low labeling index (≤10%); solid line, high labeling index (> 10%). The difference between the two curves was not statistically different.

**Table 4** Prognostic relevance of surgical treatment in cell kinetic subgroups of patients

| *LI* | *No. of cases* | *Probability (%) at 12 mo* | | | | | |
|---|---|---|---|---|---|---|---|
| | | *FFP* | | | *Survival* | | |
| | | *RS** | *nonRS*** | *p* | *RS* | *nonRS* | *p* |
| Low | 16 | 58 | 25 | ns | 100 | 63 | 0.09 |
| High | 19 | 25 | 8 | ns | 38 | 32 | ns |

* RS: radical surgery
** nonRS: nonradical surgery

In conclusion, when type of surgery and cell kinetics were taken into consideration, 100% of the patients with slowly proliferating and radically resected hepatic metastases had a probability of 12-month survival. Patients with rapidly proliferating tumors, regardless of treatment, had the worst prognosis and patients with unresectable but low LI tumors had an intermediate prognosis.

### DISCUSSION

During the last two decades, there has been an increasing trend to treat hepatic metastases from colorectal cancer. In particular liver resection has become an accepted treatment. However, the criteria for classification and staging, indications for treatments and evaluation of results are still controversial. In fact, a basic uncertainty still exists about the most relevant prognostic factors, in spite of attempts to better define histologic, anatomic, clinical and functional parameters, which could have a real impact on prognosis[11].

The potentials of a biologic classification based on serum and tissue markers to give additional or alternative information of clinical relevance have been taken into consideration for some tumor types[21–26].

The results of the present study indicate that, as observed for primary and metastatic lesions of other tumor types[27,28], cell kinetics of hepatic metastases from colorectal carcinoma, as expressed by $^{3}$H–thymidine LI, widely varies from patient to patient. The cell kinetic variable is largely independent of patient's characteristics, sex and age, or liver function markers of clinical relevance, alkaline phosphatase and CEA, and only slightly related to the degree of liver replacement in synchronous metastases.

Notwithstanding the relatively small number of patients studied and the short follow-up, the outcome of this study is indicative of the prognostic relevance of cell kinetics. In fact, cell kinetics is an important discriminant of FFP and even more of overall survival. Moreover, the cell kinetic variable appears to be a further discriminant of long-term clinical outcome in subgroups of patients defined according to one of the most important prognostic factors, i.e. resectability of metastases.

If these findings are confirmed on a larger series of patients, cell kinetics will be used as an important marker of risk, and its consideration, in association with the other factors, will be mandatory for an accurate prognostic definition and a correct evaluation of the actual effect of different treatments and important in planning treatment protocols.

### *References*

1. Gennari, L., Doci, R., Bozzetti, F., Veronesi, U. (1982). Proposal for a clinical classification of liver metastases. *Tumori*, **68**, 443–449.
2. Gennari, L., Doci, R., Bozzetti, F., Bignami, P. (1985). Proposal for staging liver metastases. In: Hellmänn K., Eccles S.A., eds. Treatment of Metastases. Problems and Prospects. London: Taylor and Francis, 37–40.
3. Bengtsson G., Carlsson G., Hafstrom L., Jonsson P. (1981). Natural history of patients with untreated liver metastases from colorectal cancer. *Am. J. Surg.*, **141**, 586–589.
4. Dahl, E.P., Fredlund, P.E., Tylen, U., Bengmark, S. (1981). Transient hepatic dearterialization followed by regional intra-arterial 5- fluorouracil infusion as treatment for liver tumors. *Ann. Surg.*, **193**, 82–88.
5. Pettavel, T., Leyvraz, S., Douglas, P. (1984). The necessity for staging liver metastases and standardizing treatment-response criteria. The case of secondaries of colo-rectal origin. In: van de Velde C.J.H., Sugarbaker P.H., eds. Liver Metastases. Basic Aspects, Detection and Management. Dordrecht: Martinus Nijhoff, 154–168.
6. Bengmark, S., Rosengren, K. (1974). Angiographic study of the liver after ligation of the hepatic artery in man. *Am. J.Surg*, **119**, 620–624.
7. Rappaport, A.H., Burleson, R.L. (1970). Survival of patients treated with systemic fluorouracil for hepatic metastases. *Surg. Gynecol. Obstet.*, **130**, 773–777.
8. Wood, C.B., Gillis, C.R., Blumgart, L.H. (1976). A retrospective study on the natural history of patients with liver metastases from colorectal cancer. *Clin. Oncol.*, **2**, 285–288.
9. Lahr, C.J., Soong, S.J.., Cloud, G., Smith, J.W., Urist, M.M., Balch, C.M. (1983). A multifactorial analysis of prognostic factors in patients with liver metastases from colorectal carcinoma. *J. Clin. Oncol.*, **1**, 720–726.

10. Fortner, J.G., Silva, J.S., Golbey, R.B., *et al.* (1984). Multivariate analysis of a personal series of 247 consecutive patients with liver metastases from colorectal cancer. I. Treatment by hepatic resection. *Ann. Surg.*, **199**, 306–316.
11. Hughes, S.K., Sugarbaker, P.H. (1987). Resection of the liver for metastatic solid tumors. In: Steven A. Rosenber G. eds. Treatment of metastatic cancer. Philadelphia: JB Lippincott Company, 130–142.
12. Bleiberg, H., Galand, P. (1976). In vitro autoradiographic determination of cell kinetic parameters in adenocarcinomas and adjacent healthy mucosa of the human colon and rectum. *Cancer Res.*, **36**, 325–328.
13. Deschner, E.E., Winawer, S.J., Long, F.C., Boyle, C.C. (1977). Early detection of colonic neoplasia in patients at high risk. *Cancer*, **40**, 2625–2631.
14. Deschner, E.E., Maskens, A.P. (1982). Significance of the labeling index and labeling distribution as kinetic parameters in colorectal mucosa of cancer patients and DMH treated animals. *Cancer*, **50**, 1136–1141.
15. Bleiberg, H., Buyse, M., Van Den Heule, B., Galand, P. (1984). Cell cycle parameters and prognosis of colorectal cancer. *Eur. J. Cancer Clin.. Oncol.*, **20**, 391–396.
16. Bleiberg, H., Buyse, M., Galand, P. (1985). Cell kinetic indicators of premalignant stages of colorectal cancer. *Cancer*, **56**, 124–129.
17. Kanemitsu, T., Koike, A., Yamamoto, S. (1985). Study of the cell proliferation kinetics in ulcerative colitis, adenomatous polyps and cancer. *Cancer*, **56**, 1094–1098.
18. Terpstra, O.T., van Blankenstein, M., Dees, J., Eilers, G.A.M. (1987). Abnormal pattern of cell proliferation in the entire colonic mucosa of patients with colon adenoma or cancer. *Gastroenterology*, **92**, 704–708.
19. Silvestrini, R., Daidone, M.G., Di Fronzo, G., Morabito, A., Valagussa, P., Bonadonna, G. (1986). Prognostic implication of labeling index versus estrogen receptors and tumor size in node-negative breast cancer. *Breast Cancer Res. Treat.*, **7**, 161–169.
20. Peto, R., Pike, M.C., Armitage, P., Breslow, N.E., Cox, D.R., Howard, S.V., Mantel, N., McPherson, K., Peto, S., Smith, P.G. (1977). Design and analysis of randomized clinical trials requiring prolonged observation of each patient. II. Analysis and examples. *Br. J. Cancer*, **35**, 1–39.
21. Bosl, G.J., Geller, N.L., Cirrincione, C., Vogelzang, N.J., Kennedy, B.J. *et al.* (1983). Multivariate analysis of prognostic variables in patients with metastatic testicular cancer. *Cancer Res.*, **43**, 3403–3407.
22. Meyer, J.S., Friedman, E., McCrate, M., Bauer, W.C. (1983). Prediction of early course of breast carcinoma by thymidine labeling. *Cancer*, **51**, 1879–1886.
23. Hagberg, H., Gimelius, B., Gronowitz, S., Killander, A., Kallander, C., Schroder, T. (1984). Biochemical markers in non-Hodgkin's lymphomas stages III and IV and prognosis: A multivariate analysis. *Scand. J. Haematol.*, **33**, 59–67.
24. Lenner, P., Roos, G., Johansson, H., Lindh, J., Dige, U. (1987). Non-Hodgkin Lymphoma. Multivariate analysis of prognostic factors including fraction of S-phase cells. *Acta. Oncol.*, **26**, 179–193.
25. Costa, A., Silvestrini, R., Bonadonna, G., Valagussa, P., Verderio, L., *et al.* (1988). Prognostic relevance of cell kinetics in non-Hodgkin's lymphomas. *Proceedings of Asco.*, **7**, abstr. 917.
26. Kallioniemi, O.P., Punnonen, R., Mattila, J., Lehtinen, M., Koivula, T. (1988). Prognostic significance of DNA index, multiploidy and S- phase fraction in ovarian cancer. *Cancer*, **61**, 334– 339.
27. Meyer, J.S. (1982). Potential value of cell kinetics in management of cancer of unknown origin. *Semin. Oncol.*, **9** 513–516.
28. Silvestrini, R., Daidone, M.G., Costa, A., Sanfilippo, O. (1985). Cell kinetics and in vitro chemosensitivity as a tool for improved management of patients. *Eur. J. Cancer*, **21**, 371–378.

### INVITED COMMENTARY

Using modern diagnostic techniques and generally accepted criteria for resection, it appears that resection of colorectal liver cancer will cure about 25% of the patients and that the incidence of relapse is approximately 50% in both the liver and the extrahepatic tissues[1–3]. In patients with irresectable hepatic metastases from colorectal cancer, palliative therapy, such as hepatic arterial infusion of fluoropyrimidines, may sometimes appear to give long-term prolongation of survival. However, no mode of palliative treatment has been shown to prolong survival in groups of patients. Although it is a clinical impression that some patients respond favourably to cytostatic treatment whereas others do not, it has not been possible to correlate survival with any pre-treatment characteristic other than the interval between diagnosis and therapy[4]. It is clear that improved detection (staging) and better grading of hepatic and extrahepatic disease is important for improved understanding and management of patients with colorectal liver cancer.

Therefore the grading method described by Silvestrini *et al.*, is of great interest. Their finding that the LI value was a prognostic determinant in patients with resectable liver metastases suggests that cell kinetic analysis may give prognostic information not available by other means. The observation that the median LI values were similar in patients with resectable and irresectable disease is potentially more important because it does not seem to correlate with well established determinants of prognosis in patients with colorectal liver cancer. However, I believe that the prognostic relevance of cell kinetic analysis is unclear because resectability (as opposed to irresectability) has been shown to be a very strong determinant of survival in untreated patients[5]. At any rate, interpretation should be cautious because the authors did not define "resectability"; for instance, it is not stated if, or to what extent, the number and localization of liver tumours influenced the decision to resect or not. Another word of caution comes from studies in patients with primary colorectal cancer in whom S phase duration and labelling index appear unrelated to prognosis[6].

It appears that the presented method has a potential for better stratification and for better selection of treatment of patients with colorectal liver cancer. As stressed by the authors themselves, it must be remembered, however, that the number of patients was small and that the follow-up was short. Further studies, with other or complementary staging systems, are necessary and will be awaited with interest, especially if combined with cell kinetic analysis of the primary tumour.

### *References*

1. Ekberg, H., Tranberg, K.-G., Andersson, R., Lundstedt, C., Hägerstrand, I., Ranstam, J. and Bengmark, S. (1987) Determinants of survival in liver resection for colorectal secondaries. *Br. J. Surg.*, **73**, 727–731.
2. Ekberg. H., Tranberg. K.-G., Andersson. R., Lundstedt. C., Hägerstrand. I., Ranstam. J. and Bengmark. S. (1987) Pattern of recurrence in liver resection for colorectal secondaries. *World J. Surg.*. **11**, 541–547.
3. Greenway. B. (1988) Hepatic metastases from colorectal cancer: resection or not. *Br. J. Surg.*, **75**, 513–519.
4. Bengmark. S. and Tranberg. K.-G. (1986) Chemotherapy (systemic and infusion) in the management of primary and secondary tumours. In: Clinical Surgery International, vol. 12, Bengmark S., Blumgart L.H., eds. Liver Surgery. London: Churchill Livingstone 35–50.
5. Wood, C.B., Gillis, C.R. and Blumgart, L.H. (1975) patient survival related to the extent of liver metastases from colorectal cancer. *Bull. Soc. Intern. Chir.*. **5**, 405–407.
6. Bleiberg, H., Buyse, M., van den Heule, B. and Galand, P. (1984) Cell cycle parameters and prognosis of colorectal cancer. *Eur. J. Cancer. Clin. Oncol.*. **20**, 391–396.

Karl-G Tranberg
Lund University
Lund, Sweden

